# Potential Anti-aging Components From *Moringa oleifera* Leaves Explored by Affinity Ultrafiltration With Multiple Drug Targets

**DOI:** 10.3389/fnut.2022.854882

**Published:** 2022-05-10

**Authors:** Yongbing Xu, Guilin Chen, Mingquan Guo

**Affiliations:** ^1^Key Laboratory of Plant Germplasm Enhancement and Specialty Agriculture, Wuhan Botanical Garden, Chinese Academy of Sciences, Wuhan, China; ^2^College of Life Sciences, University of Chinese Academy of Sciences, Beijing, China; ^3^Sino-Africa Joint Research Center, Chinese Academy of Sciences, Wuhan, China; ^4^Innovation Academy for Drug Discovery and Development, Chinese Academy of Sciences, Shanghai, China

**Keywords:** *Moringa oleifera*, anti-aging, affinity ultrafiltration, collagenase, elastase, hyaluronidase, LC-MS

## Abstract

*Moringa oleifera* (*M. oleifera*), widely used in tropical and subtropical regions, has been reported to possess good anti-aging benefits on skincare. However, the potential bioactive components responsible for its anti-aging effects, including anti-collagenase, anti-elastase, and anti-hyaluronidase activities, have not been clarified so far. In this study, *M. oleifera* leaf extracts were first conducted for anti-elastase and anti-collagenase activities *in vitro* by spectrophotometric and fluorometric assays, and the results revealed that they possessed good activities against skin aging-related enzymes. Then, multi-target bio-affinity ultrafiltration coupled to high-performance liquid chromatography-mass spectrometry (AUF-HPLC-MS) was applied to quickly screen anti-elastase, anti-collagenase, and anti-hyaluronidase ligands in *M. oleifera* leaf extracts. Meanwhile, 10, 8, and 14 phytochemicals were screened out as the potential anti-elastase, anti-collagenase, and anti-hyaluronidase ligands, respectively. Further confirmation of these potential bioactive components with anti-aging target enzymes was also implemented by molecule docking analysis. In conclusion, these results suggest that the *M. oleifera* leaves might be a very promising natural source of anti-aging agent for skincare, which can be further explored in the cosmetics and cosmeceutical industries combating aging and skin wrinkling.

## Introduction

Skin aging is one of the visible precursors of aging and mainly caused by oxidative stress and the enhanced activation of proteolytic enzymes, such as elastase, collagenase, and hyaluronidase, which belong to the matrix metalloproteinases (MMPs) (1). Hyaluronic acid performs multiple functions in the skin, including maintaining moisture and promoting the mechanical elasticity and flexibility of the skin. Moreover, elastin plays an important role in maintaining the elasticity of the skin. The degradation of elastin is the main reason for skin aging to produce sagging skin and fine wrinkles. In addition, collagen, which is widely found in the extracellular matrix, plays a pivotal role in maintaining the flexibility, strength, and elasticity of the skin (2). However, hyaluronidase, elastase, and collagenase could degrade hyaluronic acid, elastin, and collagen, respectively. Therefore, hyaluronidase, elastase, and collagenase inhibitors are potential bioactive ingredients since they have antiaging and anti-wrinkle activities on the skin.

*Moringa oleifera* (*M. oleifera*) is native to India, and it is widely planted and used in tropical and subtropical areas in recent decades, due to its high nutritive values in proteins, amino acids, fats, minerals, and vitamins (3). In contrast, the complex and diverse chemical components including flavonoids, phenolic acids, glucosinolates, and nitrile glycosides in *M. oleifera* lead to its numerous pharmacological activities, for example, antioxidant (4), anti-inflammatory (5), antibacterial (6), anticancer (7), hypoglycemic (8), and hypolipidemic (9) activities. Therefore, *M. oleifera* has received increasing attention in recent years. In the meantime, a large number of studies indicated that *M. oleifera* also possessed anti-aging properties. For instance, *M. oleifera* leaf extracts displayed anti-aging activity by improving oxidative stress resistance and nutrient-sensing pathways (10), extending the life span, and improving stress tolerance of *Caenorhabditis elegans* (11). Moreover, it was also found that the *M. oleifera* leaf extracts observably decreased age-related neurodegeneration in the old treated rat model (12), and its cream restored skin vitality and reduced skin aging (13). However, the specific bioactive ingredients in *M. oleifera* leaf extracts that contributed most to its conspicuous antiaging activity remain unexplored until now. Therefore, it is of great necessity to unravel the prominent anti-aging constituents in the *M. oleifera* leaves.

At present, an integrative strategy combining affinity ultrafiltration with LC-MS based on the interactions between small bioactive molecules and their correlative target enzymes was developed, which, thereafter, made the high-throughput screening and rapid identification of these bioactive components possible. For example, 7, 10, 6, and 7 components belonging to flavonoids and lignans in *Podophyllum sinense* were screened out as the potential Topo I, Topo II, COX-2, and ACE2 ligands, respectively (14). In addition, 10 and 14 potential bioactive constituents in *M. oleifera* leaf extracts showed high binding abilities to pancreatic lipase and α-glucosidase, respectively (15). Furthermore, 16 potential bioactive ligands targeting 5-LOX from Zi-shen pill extract were selected using the affinity ultrafiltration approach (16).

Until now, the antiaging activities of *M. oleifera* leaves, especially elastase and collagenase inhibitory activities, have not been reported yet. In this study, we aimed to explore the prominent anti-aging activities of *M. oleifera* leaves and screened out the correlated potential anti-aging multi-target components. To achieve this, the *in vitro* elastase and collagenase enzyme inhibitory assays were first conducted to evaluate the activities of *M. oleifera* leaf extracts. Then, affinity ultrafiltration coupled with LC-MS was employed to fish out the potential anti-aging components in *M. oleifera* leaves, and the subsequent verification was further implemented with molecule docking analysis. Overall, this study could provide molecular evidence for *M. oleifera* leaves as a natural source of anti-aging agents. At the same time, it will further facilitate its better application and development as a functional food or skincare product in the near future.

## Materials and Methods

### Plant Materials

The fresh leaves of *M. oleifera* were collected from a farm in Machakos County, Kenya. The specimen was authenticated by Professor Guangwan Hu, a senior botanist of Wuhan Botanical Garden, Chinese Academy of Sciences, and stored in the herbarium of the Key Laboratory of Plant Germplasm Enhancement and Specialty Agriculture with the voucher specimen numbers (No. 2018001). The sun-dried leaves were packed in sealed polyethylene bags and stored in the refrigerator at 4°C until further use.

### Chemicals and Reagents

The collagenase was supplied by Shanghai Yuanye Biotechnology Co., Ltd. (Shanghai, China). The elastase was obtained from Beijing Coolaber Technology Co., Ltd. (Beijing, China). The hyaluronidase from bovine testes and the fluorogenic MMP 2 substate (N-Succinyl-Ala-Ala-Ala-*p*-nitroanilide) were bought from Sigma-Aldrich (St Louis, MO, United States). The 10 kDa cutoff centrifugal ultrafiltration filters (YM-10) were purchased from Millipore Co., Ltd. (Bedford, MA, United States). The membranes (0.22 μm) were bought from Tianjin Jinteng Experiment Equipment Co., Ltd. (Tianjin, China). Epigallocatechin gallate was bought from Shanghai Meryer Chemical Technology Co., Ltd. (Shanghai, China). Acetonitrile (ACN) and formic acid (FA) of HPLC grade were purchased from TEDIA Company Inc. (Fairfield, Ohio, USA). The ultrapure water for LC-MS analysis and pure water for the sample process were prepared using the EPED water system (Nanjing EPED Technology Development Co., Ltd, Nanjing, China). All other chemicals were of analytical grade and supplied by Sinopharm Chemical Reagent Co., Ltd. (Shanghai, China), including methanol (MeOH), hydrochloric acid (HCl), sodium dihydrogen phosphate (NaH_2_PO_4_), dibasic sodium phosphate (Na_2_HPO_4_), and ascorbic acid.

### Preparation of Sample Extracts

For the sample preparation, first, 100 g dried powders of *M. oleifera* leaves were accurately weighed, then soaked with 800 mL 90% ethanol overnight, and later extracted with ultrasound for 30 min. Then, the extracts were filtered, and the residues were re-extracted with 90% ethanol. Then, the above extraction process was repeated twice. After the extraction, the supernatants were combined and concentrated to dryness using a rotary evaporator under a vacuum. The dried extracts were deposited in the refrigerator prior to further use.

### Determination of Collagenase Inhibitory Activity

The inhibitory activity of *M. oleifera* leaf extracts on collagenase was measured using a slightly modified spectrofluorimetric method described by Stavropoulou et al. (17). In brief, collagenase was added to Tris–HCl buffer (50 mM, pH 7.8) to obtain a concentration of 2 units/mL, and the fluorogenic metalloproteinase-2 (MMP2) substrate (MCA-Pro-Leu-Ala-Nva-DNP-Dap-Ala-Arg-NH2) was prepared at the concentration of 25 μM by being dissolved in buffer. Then, the samples (20 μL) were incubated at 37 °C with collagenase (20 μL) and Tris–HCl buffer (60 μL) in a black 96-well plate. After being kept in darkness for 15 min, the substrate solution (100 μL) was added to trigger the reaction, and the mixture solution was incubated at 37 °C for 30 min in darkness. Finally, the fluorescence values were recorded at the excitation wavelength of 320 nm and the emission wavelength of 405 nm using a multifunctional microplate reader (Tecan Infinite M200 PRO, TECAN, Männedorf, Switzerland). EGCG (6.25–200 μg/mL) was performed as the positive control, and all the samples were tested in triplicate. The inhibition rate of *M. oleifera* leaf extracts on collagenase was computed according to the following formula:


$$\begin{array}{l} \text{Inhibition rate }\left(\%\right)=\frac{\text{RF}{\text{U}}_{100}\;-\;\text{RF}{\text{U}}_{\text{sample}}}{\text{RF}{\text{U}}_{100}}\times 100 \end{array}$$

where RFU_100_ is the fluorescence value of 100% enzyme activity control group, RFU_sample_ is the fluorescence value of the tested sample or positive control, and the half inhibitory concentration (IC_50_) was acquired when the collagenase was inhibited by 50% under assay conditions.

### Determination of Elastase Inhibitory Activity

The inhibitory activity of *M. oleifera* leaf extracts on elastase was determined using N-succinyl-Ala-Ala-Ala-*p*-nitroanilide as the substrate (18). In brief, 10 μL of sample solution was mixed with 90 μL of Tris–HCl buffer solution (50 mM, pH = 7.8) and 20 μL of elastase solution (0.5 units/μL), and the mixture solution was incubated for 15 min in a 96-well plate at 37 °C in darkness. Later, 80 μL of the substrate N-succinyl-Ala-Ala-Ala-*p*-nitroanilide (2 mM, dissolved in Tris–HCl buffer solution) was added to start the reaction. After incubation for 30 min at 37 °C in darkness, the absorbance was monitored at 405 nm. Meanwhile, ascorbic acid (0.125-2 mM) was used as the positive control, and each sample was tested in triplicate. The inhibitory activity of *M. oleifera* leaf extracts on elastase was expressed as 50% inhibitory concentration (IC_50_). The inhibition rate was calculated according to the following formula:


$$\begin{array}{l} \text{Inhibition rate }\left(\%\right)=(1-\frac{\text{Ab}{\text{s}}_{\text{sample}}\;-\;\text{Ab}{\text{s}}_{\text{sample}-\text{blank}}}{\text{Ab}{\text{s}}_{\text{control}}\;-\;\text{Ab}{\text{s}}_{\text{control}-\text{blank}}})\times 100 \end{array}$$

where Abs_sample_ is the absorbance of sample group with elastase, Abs_sample−blank_ is the absorbance of sample group without elastase, Abs_control_ is the absorbance of 100% elastase, and Abs_control−blank_ is the absorbance of the reagent blank without elastase.

### Affinity Ultrafiltration Procedures

The potential anti-aging components in *M. oleifera* leaves exhibiting different binding affinities with elastase, collagenase, and hyaluronidase were screened out by affinity ultrafiltration. The screening procedures were conducted according to our previous method with some modifications (15, 19), which mainly includes three major steps, namely, incubation, ultrafiltration, and analysis. In brief, the *M. oleifera* leaf extracts were weighed accurately and fully dissolved in Tris–HCl buffer solution (pH = 7.8, for elastase and collagenase) or PBS buffer solution (pH = 5.35, for hyaluronidase) at the final concentration of 8 mg/mL. Later, 100 μL of prepared sample solution was mixed with 20 μL of elastase (10 U), 40 μL of collagenase (2 U), or 40 μL of hyaluronidase (60 U) in a 1.5 mL EP tube and then incubated at 37 °C for 40 min in the water bath. At the same time, for the negative control group, the incubation conditions for the inactivated elastase, collagenase, or hyaluronidase, which was obtained in boiling water for 15 min, were the same as active enzymes. After incubation, the mixtures were ultra-filtrated through a 10 KD cutoff ultrafiltration membrane (Millipore, 0.5 mL) by centrifuging at 10,000 rpm for 10 min. Then, the membranes were eluted with 200 μL Tris–HCl or PBS buffer solutions 3 times to remove the unbound ligands. Subsequently, the ligands with specific bindings to elastase, collagenase, or hyaluronidase were released from the complexes by being incubated with 200 μL of 90% *(v/v)* MeOH-H_2_O for 10 min at room temperature and followed by centrifugation at 10,000 rpm for 10 min. This step was repeated three times. Later, the filtrates above were collected and combined. Finally, they were dried using a nitrogen blower and then dissolved in 50 μL of methanol for the HPLC-UV-ESI-MS/MS analysis.

By comparing the varieties of peak areas in the chromatograms of active and inactive enzymes, specific bindings to elastase, collagenase, and hyaluronidase were defined with the binding degree (BD) using the following formula (20):


$$\begin{array}{l} \text{Binding degree }(\%)=\left(\frac{{\text{A}}_{\text{a}}\;-\;{\text{A}}_{\text{b}}}{{\text{A}}_{\text{a}}}\right)\times 100\% \end{array}$$

where *A*_a_ and *A*_b_ are the peak areas in the chromatograms of active and inactive elastase, collagenase and hyaluronidase enzymes, respectively. If the peak area in the active enzyme group is bigger than that in the inactive enzyme group, the compound is considered as potentially active ligands.

### HPLC-UV/ESI-MS/MS Analysis

The HPLC-UV/ESI-MS/MS analysis was performed using a TSQ Quantum Access MAX mass spectrometer in the negative mode, which was connected with a Thermo Accela 600 series HPLC system (Thermo Fisher Scientific, San Jose, CA, USA). The chromatographic separation of samples was carried out using a Waters Symmetry RP-C18 column (250 mm × 4.6 mm, 5 μm) at 25 °C. The mobile phases consisted of 0.1% (*v/v*) formic acid–water (A) and acetonitrile (B). The HPLC elution procedures were optimized as follows: 0–30 min, 8–30% B; 30–40 min, 30–95% B. The injection volume of all the samples was 10 μL, the flow rate was set at 800 μL/min, and the wavelength was monitored at 280 nm. Moreover, the optimized parameters of the MS instrument were implemented as follows: capillary temperature of 350 °C; vaporizer temperature of 300 °C; spray voltage of 3,000 V; sheath gas (nitrogen, N_2_) of 40 psi; auxiliary gas (N_2_) of 10 psi; and mass range (*m/z*) of 100–1,500. The mass spectrum data were obtained in the full-scan and the data-dependent mode and analyzed by the Thermo Xcalibur ChemStation (Thermo Fisher Scientific).

### Molecular Docking Analysis

Molecular docking analysis between potential ligands in *M. oleifera* leaf extracts and elastase, collagenase, and hyaluronidase were performed by the AutoDock Vina 1.5.6 and Discovery Studio 4.5 Client. The analytical steps generally consisted of the measurement of the binding modes between the proteins and the small ligands, assessment of the scoring system, and the calculation of docking results according to our previous study (15). In brief, the crystal structures of elastase (PDB: 1U4G), collagenase (PDB: 1CGL), and hyaluronidase (PDB: 1FCV) were downloaded from the RCSB Protein Data Bank (www.rcsb.org). The three-dimensional (3D) structures of the ligands with the lowest energies were obtained by ChemBio3D Ultra. Subsequently, the 3D structures of ligands and receptors were processed by removing the water molecules, adding the hydrogen atoms, calculating the charge, and so on by AutoDock Tools. Later, the docking active sites of elastase, collagenase, and hyaluronidase were obtained by Discovery Studio 4.5 Client. Thereinto, the coordinates of the active sites of elastase were (X: 17.554; Y: 28.659; Z: −4.625), collagenase (X: 23.679; Y: 44.947; Z: −8.569), and hyaluronidase (X: 4.447; Y: 29.902; Z: −9.110), respectively. Besides, the grid box was centered on the active sites of the receptors with a dimension size of 60 Å × 60 Å × 60 Å. Finally, molecular docking analysis between ligands and receptors was performed with 50 independent runs of the genetic algorithm by AutoDock Tools with other default parameters. In addition, EGCG was used as a positive control against collagenase and hyaluronidase enzymes, and ascorbic acid was applied as a positive drug against elastase enzyme.

### Statistical Analysis

In this study, the half inhibitory concentration (IC_50_) was calculated by nonlinear regression analysis and represented as means ± standard deviation (SD) employed using SPSS 22.0 (IBM Corp., New York, USA).

## Results

### *In vitro* Collagenase and Elastase Inhibitory Activities

**Figure 1:**
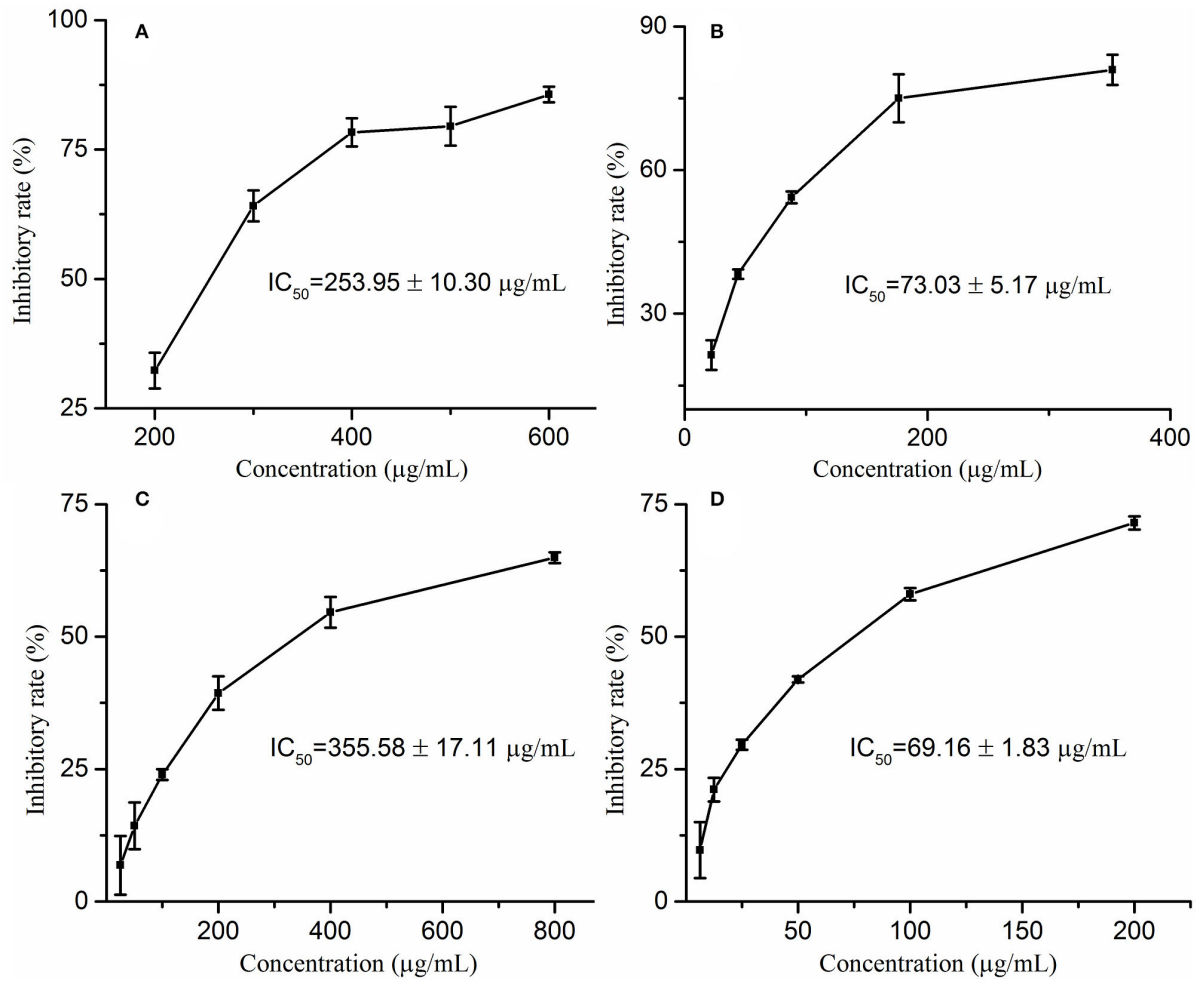
The inhibitory rates of *Moringa oleifera* leaf extracts on elastase **(A)** and collagenase **(C)** as well as their corresponding positive drugs ascorbic acid **(B)** on elastase, EGCG **(D)** on collagenase.

Wrinkles are one of the characteristics of aging, and the degradation of elastin and collagen is the main cause of wrinkles. Moreover, elastase and collagenase play a pivotal role in the degradation of elastin and collagen, respectively. Therefore, exploring natural elastase and collagenase inhibitors has become one of the effective ways to delay aging. In this study, the anti-aging activities of *M. oleifera* leaf extracts were evaluated by determining the inhibitory activities against collagenase and elastase *in vitro*. On the one hand, this study reveals that *M. oleifera* leaf extracts exhibited a promising anti-collagenase effect in a concentration-dependent manner with a 50% inhibition value (IC_50_ value) at 355.58 ± 17.11 μg/mL, whereas the IC_50_ value of the positive drug epigallocatechin gallate (EGCG) was 69.16 ± 1.83 μg/mL (Figures 1A,B). On the other hand, it is clear from Figures 1C,D that *M. oleifera* leaf extracts showed considerable elastase inhibitory activity with an IC_50_ value of 253.95 ± 10.30 μg/mL, while the positive control ascorbic acid showed an IC_50_ value of 73.03 ± 5.17 μg/mL.

### Screening for the Potential Elastase, Collagenase, and Hyaluronidase Ligands in *M. oleifera*

**Figure 2:**
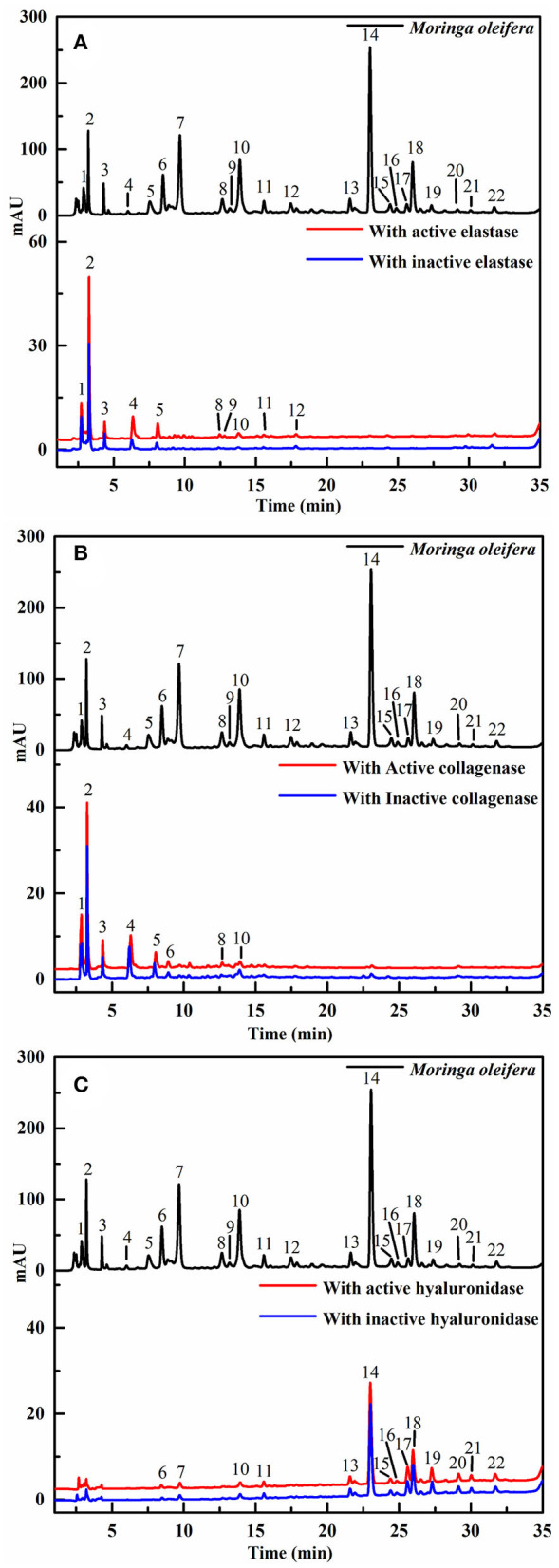
The high-performance liquid chromatography (HPLC) profiles of the chemical components in *M. oleifera* were monitored after affinity ultrafiltration at 280 nm. The black line represents HPLC chromatograms of *M. oleifera* leaf extracts without ultrafiltration; the red line and blue line represent the leaf extract of *M. oleifera* with activated and inactivated elastase **(A)** collagenase **(B)** and hyaluronidase **(C)** respectively.

Recently, affinity ultrafiltration coupled with high-performance liquid chromatography-mass spectrometry (AUF-HPLC-MS) technology has been successfully used to find out the bioactive ingredients in complex samples (21, 22). In this study, the AUF-LC-MS method was employed to screen the potential anti-aging components against elastase, collagenase, and hyaluronidase. After incubation with elastase, collagenase, and hyaluronidase for affinity ultrafiltration, the bound ligands in *M. oleifera* leaves were released and analyzed by LC-MS. As shown in Figure 2, it was observed that 10, 8, and 14 phytochemicals from *M. oleifera* leaf extracts exhibited differential bindings to the elastase, collagenase, and hyaluronidase enzymes, respectively. The specific BD of each compound is displayed in Table 1. Among them, 4-caffeoylquinic acid (Peak 8) possessed the highest specific BD (49.15%) for collagenase enzyme, followed by quinic acid (37.18%), vicenin-2 (26.92%), etc. Similarly, 4-caffeoylquinic acid (Peak 8) also displayed the highest specific BD (62.34%) for elastase enzyme, followed by vicenin-2 (59.49%), glucomoringin (59.13%), etc. In addition, 3-caffeoylquinic acid (Peak 6) exhibited the strongest specific BD (36.75%), followed by kaempferol 3-*O*-rutinoside (27.11%), 3-coumaroylquinic acid (21.87%), and so on.

**Table 1:** The binding degree (BD) and the affinity ultrafiltration coupled with high-performance liquid chromatography-mass spectrometry (AUF-LC/MS) data of the bioactive compounds bound to elastase, collagenase, and hyaluronidase enzymes from *Moringa oleifera* leaf extracts.

**Peak No**.	**RT^a^ (min)**	**BD^b^ (%)**			**[M-H]^−^**	**Characteristic fragment (*m/z*)**	**Identification**
		**Ela^c^**	**Col^d^**	**Hya^e^**			
1	2.88	3.43	20.44	-	341	341, 179, 119, 113, 89	Sucrose
2	3.21	36.76	37.18	-	191	191, 173, 127, 93, 85	Quinic acid
3	4.29	10.62	7.12	-	290	290, 200, 170, 154, 128	*N*-Fructosyl pyroglutamate
4	6.00	58.00	8.90	-	326	164, 147, 101	Methyl 4-(α-L-rhamnopyranosyloxy) benzylcarbamate
5	7.54	59.13	13.31	-	570	570, 328, 259, 241,97	Glucomoringin
6	8.49	-	25.83	36.75	353	191, 179, 135	3-Caffeoylquinic acid
7	9.66	-	-	21.87	337	191, 163, 119	3-Coumaroylquinic acid
8	12.65	62.34	49.15	-	353	191, 179, 173, 135	4-Caffeoylquinic acid
9	13.17	58.91	-	-	401	401, 269, 161, 113	Benzyl alcohol-hexose-pentose
10	13.88	59.49	26.91	9.53	593	593, 473, 383, 353	Vicenin-2
11	15.58	39.64	-	10.02	337	173, 163, 119	4-Coumaroylquinic acid
12	17.46	5.98	-	-	612	612, 370, 259, 97	4-(*O*-Acetyl-α-L-rhamnopyranosyloxy) benzyl glucosinolate
13	21.62	-	-	11.53	431	341, 311, 283	Apigenin 8-*C*-glucoside
14	23.04	-	-	10.60	463	463, 301, 300	Quercetin 3-*O*-glucoside
15	24.46	-	-	27.11	593	593, 285	Kaempferol 3-*O*-rutinoside
16	24.89	-	-	11.65	607	545, 505, 463, 301, 300	Quercetin-hydroxy-methylglutaroyl glycoside
17	25.62	-	-	16.63	549	505, 301, 300	Quercetin 3-*O*-(6”-malonylglucoside)
18	26.04	-	-	15.03	447	447, 285, 284, 255	Kaempferol 3-*O*-glucoside
19	27.36	-	-	14.34	477	477, 315, 314, 243	Isorhamnetin 3-*O*-glucoside
20	29.19	-	-	11.38	533	489, 285, 284	Kaempferol 3-*O*-(6”-malonylglucoside)
21	30.11	-	-	14.23	531	531, 463, 301, 300, 271	Quercetin derivative
22	31.78	-	-	13.81	515	515, 285, 284, 269, 255	Kaempferol-di-acetyl-rhamnoside

a *RT, retention time*;

b *BD, binding degree*;

c *Ela, elastase*;

d *Col, collagenase*;

e *Hya, hyaluronidase*.

### Structural Identification of the Elastase, Collagenase, and Hyaluronidase Ligands by LC-MS

**Figure 3:**
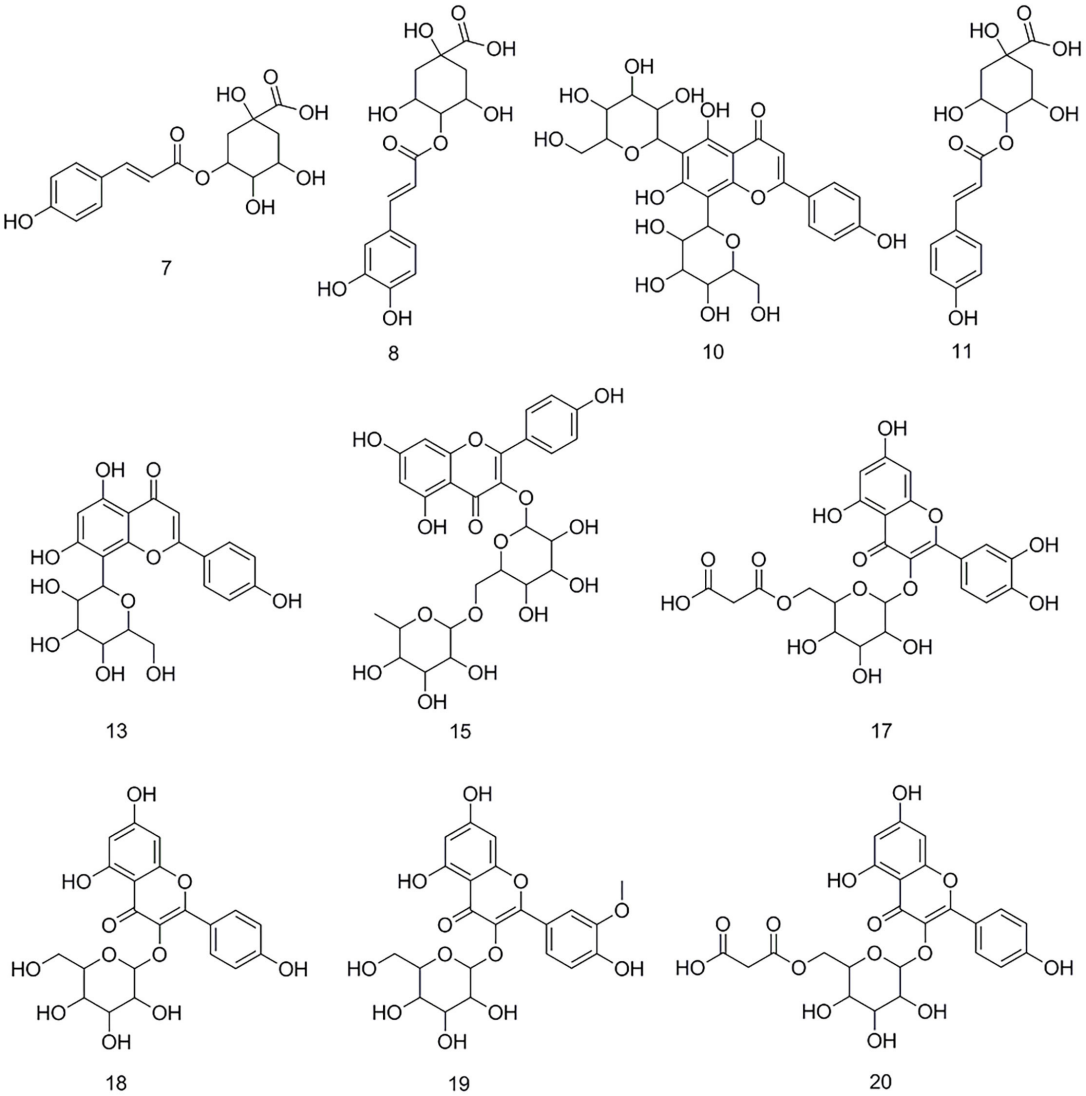
The representative bioactive compounds figured out and identified in *M. oleifera* leaf extracts.

After the affinity ultrafiltration screening procedures, 10, 8, and 14 phytochemicals in *M. oleifera* leaf extracts displayed specific bindings to elastase, collagenase, and hyaluronidase enzymes, respectively, as exhibited in Figure 2. These potential bioactive compounds were further identified and characterized by LC-MS. The details of these components of mass spectra data including retention time (RT), quasi-molecular ions ([M–H]^−^), and the characteristic MS/MS fragments are shown in Table 1. As a consequence, a total of 22 potential bioactive ligands in *M. oleifera* leaf extracts were continuously characterized and identified by comparing their mass spectra data and the fragmentation pathways with previous studies (4, 23–28). The representative bioactive ligands in *M. oleifera* leaf extracts with elastase, collagenase, and hyaluronidase enzymes are shown in Figure 3.

### Molecular Docking Analysis

**Figure 4:**
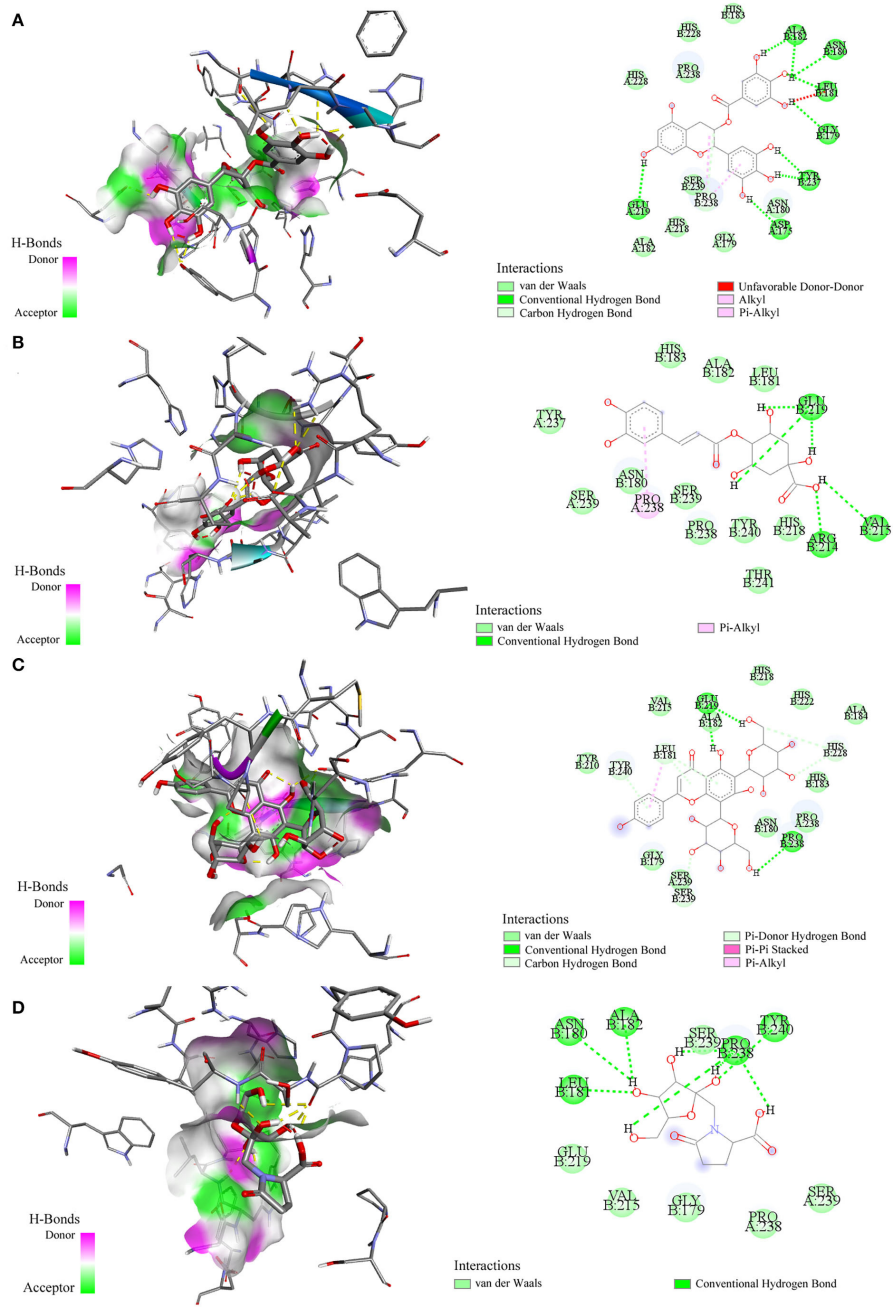
Interactions between collagenase and epigallocatechin gallate **(A)** 4-caffeoylquinic acid **(B)** vicenin-2 **(C)** and N-fructosyl pyroglutamate **(D)** by molecular docking analysis, respectively.

Molecular docking has been commonly used to evaluate the interaction between ligands and macromolecular receptors at atomic and molecular levels and predict the optimal binding situation by studying their docking energy, site of action, and key residues (29, 30). In this study, a molecular docking strategy was employed to simulate the interactions between elastase, collagenase, and hyaluronidase, and some representative bioactive compounds fished out from *M. oleifera* leaf extracts for the sake of their potential mechanism of action. The molecular docking results are displayed in Table 2. Thereinto, 4-caffeoylquinic acid (Peak 8) showed a stronger affinity to elastase with the lower binding energy (BE) of −3.93 kcal/mol and the theoretical IC_50_ value of 1.31 mM, and it was comparable with the positive drug ascorbic acid (−4.01 kcal/mol and 1.14 mM). On the contrary, sucrose displayed a lower affinity to elastase with the BE of −1.00 kcal/mol and the theoretical IC_50_ value of 183.99 mM. As regards to collagenase, 4-caffeoylquinic acid (Peak 8) also displayed a higher affinity to collagenase with the lower BE of −4.74 kcal/mol, and the theoretical IC_50_ value of 0.33 mM, which was weaker than the positive drug epigallocatechin gallate (−5.96 kcal/mol and 0.04 mM) but stronger than *N*-fructosyl pyroglutamate (Peak 3, −2.87 kcal/mol and 7.92 mM). In addition, the results of the 2D and 3D ligand-to-protein interactions between collagenase and *N*-fructosyl pyroglutamate (Peak 3), 4-caffeoylquinic acid (Peak 8), vicenin-2 (Peak 10), and epigallocatechin gallate are shown in Figure 4. With respect to hyaluronidase, 3-caffeoylquinic acid (Peak 6) exhibited a strong affinity to hyaluronidase, and its BE and theoretical IC_50_ value were computed as −4.25 kcal/mol and 0.77 mM, respectively, lower than the positive drug epigallocatechin gallate (−5.03 kcal/mol and 0.20 mM). In the meantime, vicenin-2 (Peak 10) exhibited a lower affinity to hyaluronidase with the BE of −2.02 kcal/mol and the theoretical IC_50_ value of 33.17 mM. As displayed in Tables 1, 2, it was found that descending orders of BEs of some representative potential ligands screened out from *M. oleifera* leaf extracts in the molecular docking results were consistent with their BDs in the affinity ultrafiltration results. For example, 4-caffeoylquinic acid (Peak 8) with the highest BD exhibited the strongest affinity to elastase and collagenase, possessing the most potential anti-elastase and anti-collagenase activities by molecular docking analysis. Similarly, 3-caffeoylquinic acid (Peak 6) with high BD exerted good affinity to hyaluronidase, owning potential anti-hyaluronidase effect by molecular docking analysis.

**Table 2:** The molecular docking results of the presentative active ingredients in *M. oleifera* leaf extracts and positive drugs against elastase, collagenase, and hyaluronidase, respectively.

**No**.	**Compounds**	**Protein**	**BE^a^ (kcal/mol)**	**IC_**50**_ (mM)**	**H-bond atoms^b^**
1	Sucrose	Elastase	−1.00	183.99	Ala113, Trp115, Glu141
2	*N*-Fructosyl pyroglutamate	Collagenase	−2.87	7.92	Leu181, Ala182, Pro238, Tyr240
3	3-Caffeoylquinic acid	Hyaluronidase	−4.25	0.77	Arg47, Asp111, Glu113
4	4-Caffeoylquinic acid	Elastase	−3.93	1.31	Asn112, Trp115
		Collagenase	−4.74	0.33	Arg214, Val215, Glu219
5	Vicenin-2	Elastase	−2.80	8.91	Trp115, Glu141, Glu164, Asp221
		Collagenase	−4.52	0.49	Glu219, Pro 238
		Hyaluronidase	−2.02	33.17	Arg47, Asp56, Gly58, Ser304
6	Kaempferol 3-*O*-glucoside	Hyaluronidase	−3.80	1.65	Arg47, Tyr55, Asp56, Gln98, Ser304
7	Ascorbic acid^c^	Elastase	−4.01	1.14	Asn112, Ala113, Trp115, His144, Glu164, His223
8	Epigallocatechin gallate^d^	Collagenase	−5.96	0.04	Asp175, Gly179, Asn180, Leu181, Ala182, Glu219, Tyr237
		Hyaluronidase	−5.03	0.20	Asp111, Glu113, Tyr184, Gly302

a *BE, binding energy*;

b *H-bond, hydrogen bond*;

c,d *positive drug*;

*Ala, alanine; Arg, arginase; Asn, asparagine; Asp, aspartic acid; Gln, glutamine; Glu, glutamic acid; Gly, glycine; His, histidine; Leu, leucine; Pro, proline; Ser, serine; Trp, tryptophan; Tyr, tyrosine; Val, valine*.

## Discussion

### *In vitro* Collagenase and Elastase Inhibitory Activities

Recently, plenty of studies has indicated that *M. oleifera* possesses anti-aging activity and can delay skin aging (11, 13, 31). For example, a previous study found that *M. oleifera* leaf extracts possess the most powerful life span extending property of *C. elegans* (11). However, most of them used animal test models or cell models, and there is scarcely any research at the protein molecular level. Therefore, this study determines the inhibitory activities of *M. oleifera* leaf extracts on elastase and collagenase enzymes *in vitro*. The results indicated that M. *oleifera* leaf extracts exerted considerable anti-aging effects with IC_50_ values of 253.95 ± 10.30 μg/mL against elastase and 355.58 ± 17.11 μg/mL against collagenase. In a similar study, it was found that *Eucalyptus camaldulensis* bark extract showed IC_50_ values of 343.3 ± 5.4 μg/mL against elastase and 416.3 ± 5.6 μg/mL against collagenase (32). In another study, Freitas et al. measured the elastase and collagenase inhibitory activities of *Fucus spiralis* in 11 fractions, and the inhibitory effects of different fractions on elastase were from 3.0 μg/mL to 1,000 μg/mL (IC_50_ values), whereas the inhibitory effects on collagenase were 0.037–1,000 μg/mL (IC_50_ values) (33). In addition, a previous study found that *Phyllanthus emblica* displayed IC_50_ values of 387.85 ± 8.78 μg/mL against elastase, whereas *Manilkara zapota* was 35.73 ± 0.61 μg/mL against elastase enzyme (34). Compared with those plant materials, *M. oleifera* possessed moderate anti-elastase and anti-collagenase effects. To the best of our knowledge, this is the first study to determine the anti-elastase and anti-collagenase activities of *M. oleifera* leaf extracts.

### Screening for the Potential Ligands in *M. oleifera* Against Elastase, Collagenase, and Hyaluronidase

**Figure 5:**
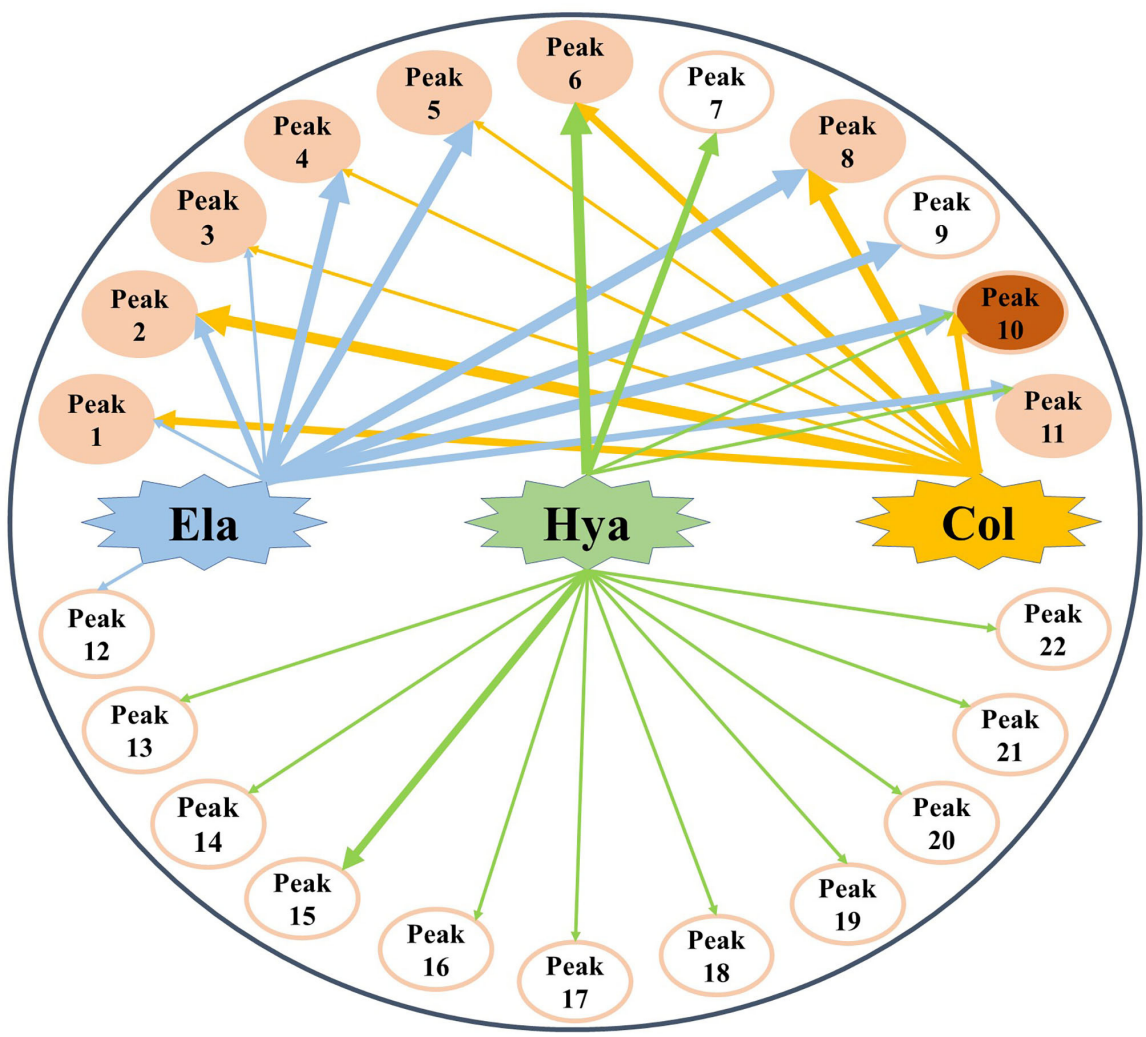
The multicomponent and multi-target network between target enzymes and their corresponding potential bioactive ligands screened out from *M. oleifera* leaf extracts. The thicknesses of lines roughly represent the binding intensity for their corresponding interactions. Ela, elastase; Hya, hyaluronidase; Col, collagenase.

Generally, the conventional strategy to screen bioactive components from the medicinal plants by enzyme inhibitory assays is both time-consuming and labor-intensive. For this purpose, affinity ultrafiltration based on the interactions between small bioactive molecules and the corresponding target enzymes coupled with high-performance liquid chromatography-mass spectrometry technology was developed in recent decades (35, 36). In this study, the AUF-HPLC/MS method was employed to fish out and identify 10, 8, and 14 potential bioactive phytochemicals in *M. oleifera* leaf extracts against elastase, collagenase, and hyaluronidase, respectively. To further investigate the underlying mechanisms of *M. oleifera* leaf extracts in the multi-constituent and multi-target manner, the potential active phytochemicals fished out from *M. oleifera* with multiple-target enzymes including elastase, collagenase, and hyaluronidase were then reconstructed as interaction network diagrams to better illuminate the relationships between the multicomponents and their corresponding target enzymes, as exhibited in Figure 5. According to the BD of each compound figured out from *M. oleifera* leaf extracts, Peaks 4, 5, 8, 9, and 10 exerted higher affinities with elastase, and they were preliminarily speculated to be the main active anti-elastase constituents. Furthermore, Peaks 2 and 8 exhibited relatively higher binding affinities with collagenase compared with other ligands, and they were inferred to be the primary anti-collagenase active ingredients. Moreover, Peak 6 displayed a stronger binding affinity to hyaluronidase, which may be the potential active anti-hyaluronidase compounds in *M. oleifera* leaf extracts. Interestingly, the major peaks in *M. oleifera* leaf extracts such as Peaks 14 and 18 were nearly undetected when subjected to bio-affinity ultrafiltration with elastase and collagenase. On the contrary, the dominant peaks including Peaks 14 and 18 were detected when subjected to bio-affinity ultrafiltration with hyaluronidase. More importantly, the results indicated that the potential active ligands of elastase and collagenase have a higher degree of overlap, while hyaluronidase is less overlapped with the other two enzymes, indicating that the active sites of elastase and collagenase may be similar, whereas the active sites of hyaluronidase may be different with elastase and collagenase. In the meantime, these shared common active phytochemicals (Peaks 1, 2, 3, 4, 5, 6, 8, 10, and 11) screened out from *M. oleifera* leaf extracts may exert synergistic effects in anti-aging activities, especially Peak 10 showed a good binding affinity with these all three enzymes. Meanwhile, those compounds with higher BDs mostly were phenolic acids and flavonoids and suggested that flavonoids and phenolic acids were the main potential antiaging components in *M. oleifera* leaf extracts. The results were similar to previous research (17, 37).

In addition, the bioactive activity of one compound is closely related to its structure. The structure-activity relationship was illuminating in this study by taking 4-caffeoylquinic acid (Peak 8) and 3-caffeoylquinic acid (Peak 6) as an example. It was reported that caffeoyl groups bound to quinic acid are important for activity (38). This may be due to the two neighboring phenolic structures as well as the α, β-conjugated unsaturated ester structure of the caffeoyl group increased the benzene ring planar conjugation. Moreover, the previous research found that quinic acid esterified with caffeoyl groups at the C-4 position showed higher anti-free radical activity compared with acylation at the C-3 or C-5 positions (39). Interestingly, in this study, 4-caffeoylquinic acid (Peak 8) showed a higher BD value than 3-caffeoylquinic acid (Peak 6) in *M. oleifera* leaf extracts with collagenase. It suggested that quinic acid esterified with caffeoyl groups at the C-4 position showed a stronger binding affinity with collagenase compared with acylation at the C-3 position.

### Molecular Docking Analysis

As exhibited in Table 1, some potential active constituents had been quickly figured out from *M. oleifera* leaf extract against elastase, collagenase, and hyaluronidase. Thereinto, 4-caffeoylquinic acid (Peak 8), quinic acid (Peak 2), and vicenin-2 (Peak 10) possessed the relatively higher BD (49.15%, 37.18%, and 26.92%) to collagenase enzyme, and *N*-fructosyl pyroglutamate (Peak 3) showed relatively lower BD (7.12%). Furthermore, 4-caffeoylquinic acid (Peak 8) also exhibited the highest specific BD (62.34%) for elastase enzyme; on the contrary, sucrose (Peak 1) possessed the lowest BD (3.43%). In addition, 3-caffeoylquinic acid (Peak 6) displayed the greatest higher BD (36.75%), whereas vicenin-2 (Peak 10) possessed the relatively lower BD (9.53%). The molecular docking results of these components and positive drugs including ascorbic acid and epigallocatechin gallate against elastase, collagenase, and hyaluronidase are displayed in Table 2, which indicated that the potential active ligands with higher BD possessed lower binding energy (BE) to enzymes and lower theoretical IC_50_ values. For example, 4-caffeoylquinic acid (Peak 8), vicenin-2 (Peak 10) and *N*-fructosyl pyroglutamate (Peak 3) possessed the BE of −4.74, −4.52, and −2.87 kcal/mol, as well as the theoretical IC_50_ values of 0.33, 0.49, and 7.92 mM, respectively. The results were consistent with their results of BD with collagenase. In addition, the main peaks such as 18 and 14 in *M. oleifera* leaf extracts were fished out by binding with hyaluronidase, while terms of elastase and collagenase were undetected. Meanwhile, the active components screened out by binding with elastase and collagenase were similar. Furthermore, the molecular docking results also supported this finding. For example, 4-caffeoylquinic acid (Peak 8) possessed the BE values of −3.93 and −4.74 kcal/mol, as well as the theoretical IC_50_ values of 1.31 and 0.33 mM with elastase and collagenase, respectively.

Moreover, these potential active components formed diverse numbers of hydrogen bonds (H-bond) with different types of amino acid residues of elastase, e.g. Asn112, Ala113, Trp115, Glu141, Glu164, and Asp221. As for collagenase, the amino acid residues formed H-bonds with these potential active constituents, including Leu181, Ala182, Arg214, Val215, Glu219, Pro238, Tyr240, and so on. Similarly, Arg47, Tyr55, Asp56, Gly58, Gln98, Asp111, Glu113, Ser304, and the like formed H-bonds with potential active ligands binding with hyaluronidase. Meanwhile, the positive drug ascorbic acid formed H-bonds with amino acid residues of Asn112, Ala113, Trp115, His144, Glu164, and His223 at elastase. 4-Caffeoylquinic acid (Peak 8) with higher BD showed the H-bonds with the acid residues of Asn112 and Trp115 at elastase. It can be inferred that the residues Asn112 and Trp115 were the key residues of elastase. Additionally, this interaction may influence the elastase structure and activity. In addition, the positive drug EGCG formed H-bonds with the residues Asp111, Glu113, Tyr184, and Gly302 of hyaluronidase, and 3-caffeoylquinic acid (Peak 6) with high special binding affinity formed H-bonds with the residues Arg47, Asp111, and Glu113 of hyaluronidase. The result suggested that the residues Asp111 and Glu113 were the potential crucial residues of hyaluronidase. Moreover, there are some other forces including the van der Waals force, Pi-Alkyl, Alkyl, and Pi-Pi stacked forces contributed to the interactions between the three enzymes and those potential active ligands in the molecular docking analysis. Taking collagenase as an example, more details about the interaction between collagenase and its potential active ligands as well as the positive drug EGCG are displayed in Figure 4. Overall, the results of molecular docking analysis were consistent with affinity ultrafiltration. Therefore, these phytochemicals screened out from *M. oleifera* leaf extracts were promising to be developed as anti-aging agents.

## Conclusion

At present, the potential bioactive phytochemicals and the underlying mechanism of *M. oleifera* leaf extracts for anti-aging activity remained elusive. In this study, the *in vitro* inhibitory assays against elastase and collagenase first confirmed that *M. oleifera* leaf extracts possessed promising anti-elastase and anti-collagenase activities. Then, 10, 8, and 14 potential bioactive phytochemicals were screened out from *M. oleifera* leaf extracts against elastase, collagenase, and hyaluronidase using the AUF-HPLC-MS approach, respectively. In addition, further verification of representative active components was employed by molecular docking analysis. To the best of our knowledge, this is the first study to systematically study the anti-elastase and anti-collagenase activities of *M. oleifera in vitro*, the potential bioactive constituents, as well as underlying mechanisms by AUF-HPLC-MS method and molecular docking analysis. In brief, the results indicated that *M. oleifera* leaf extracts exhibited an outstanding anti-aging effect, and *M. oleifera* leaves were a very promising natural source of anti-skin aging ingredients, which can be further explored in cosmetics and cosmeceutical industries combating aging and skin wrinkling.

## Data Availability Statement

The original contributions presented in the study are included in the article/supplementary material, further inquiries can be directed to the corresponding author.

## Author Contributions

YX: investigation, data curation, formal analysis, software, validation, and writing – original draft. GC: data curation, formal analysis, writing – review and editing, and funding acquisition. MG: conceptualization, methodology, resources, visualization, and project administration, supervision, writing – review and editing, and funding acquisition. All authors contributed to the article and approved the submitted version.

## Funding

This study was partly supported by the Natural Science Foundation of Hubei Province (Grant No. 2019CFB254 to GC).

## Conflict of Interest

The authors declare that the research was conducted in the absence of any commercial or financial relationships that could be construed as a potential conflict of interest.

## Publisher's Note

All claims expressed in this article are solely those of the authors and do not necessarily represent those of their affiliated organizations, or those of the publisher, the editors and the reviewers. Any product that may be evaluated in this article, or claim that may be made by its manufacturer, is not guaranteed or endorsed by the publisher.
